# Efficient HLA imputation from sequential SNPs data by transformer

**DOI:** 10.1038/s10038-024-01278-x

**Published:** 2024-08-02

**Authors:** Kaho Tanaka, Kosuke Kato, Naoki Nonaka, Jun Seita

**Affiliations:** 1 Faculty of Engineering, Kyoto University, Kyoto, Japan; 2 Advanced Data Science Project, RIKEN Information R&D and Strategy Headquarters, RIKEN, Tokyo, Japan

**Keywords:** Genetic predisposition to disease, Genetics research

## Abstract

Human leukocyte antigen (HLA) genes are associated with a variety of diseases, yet the direct typing of HLA alleles is both time-consuming and costly. Consequently, various imputation methods leveraging sequential single nucleotide polymorphisms (SNPs) data have been proposed, employing either statistical or deep learning models, such as the convolutional neural network (CNN)-based model, DEEP*HLA. However, these methods exhibit limited imputation efficiency for infrequent alleles and necessitate a large size of reference dataset. In this context, we have developed a Transformer-based model to HLA allele imputation, named “HLA Reliable IMpuatioN by Transformer (HLARIMNT)” designed to exploit the sequential nature of SNPs data. We evaluated HLARIMNT’s performance using two distinct reference panels; Pan-Asian reference panel (*n* = 530) and Type 1 Diabetes genetics Consortium (T1DGC) reference panel (*n* = 5225), alongside a combined panel (*n* = 1060). HLARIMNT demonstrated superior accuracy to DEEP*HLA across several indices, particularly for infrequent alleles. Furthermore, we explored the impact of varying training data sizes on imputation accuracy, finding that HLARIMNT consistently outperformed across all data size. These findings suggest that Transformer-based models can efficiently impute not only HLA types but potentially other gene types from sequential SNPs data.

## Introduction

The major histocompatibility complex (MHC) region, situated on the short arm of chromosome 6, exhibits a strong association with complex human traits [1]. Within this region, Human leukocyte antigen (HLA) genes are notably abundant and significantly contribute to the genetic susceptibility found therein [1]. Certain HLA alleles have been identified as risk factors for the development of severe diseases, including adverse reactions to drugs [2, 3], underscoring the importance of HLA genotyping in medical practice.

However, the direct typing of HLA alleles is challenged by the complexity of the MHC region. Techniques such as Sanger sequencing and next-generation sequencing (NGS) are the conventional methods for allele typing, but they are time-intensive, expensive, and not suitable for mass production of analysis results. Moreover, limitations in HLA gene coverage and allele resolution further complicate allele typing efforts [4, 5].

Consequently, HLA alleles are often computationally imputed using statistical models that rely on observed single nucleotide polymorphism (SNP) data, derived from ethnicity-specific reference panels [4, 6–8]. For instance, HLA*IMP utilizes the Li Stephens haplotype model [9] with SNP data from European populations [10, 11], while HLA*IMP:02 extends this approach by incorporating SNP data from multiple populations [12]. Another model, SNP2HLA, employs the imputation software package Beagle for the imputation of classical HLA alleles, achieving notable accuracy [13]. Additionally, HLA Genotype Imputation with Attribute Bagging (HIBAG) utilizes multiple expectation-maximization-based classifiers to estimate HLA allele likelihoods [14]. CookHLA, leveraging a standard hidden Markov model that integrates genetic distance as an input, utilizes Beagle v4 and v5 for its computations [15].

Despite the advancements in imputation methodologies, there remained substantial scope for enhancement, particularly regarding imputation accuracy for infrequent alleles. Since reference panels were used directly other than HIBAG, there were restrictions on the data that can be accessed from the standpoint of personal information protection, which further reduced the accuracy of the imputation.

In the realm of machine learning, significant progress has been achieved through the application of deep learning across various domains. Beyond traditional models such as Convolutional Neural Networks (CNNs) and Recurrent Neural Networks (RNNs), the Transformer model, renowned for its high accuracy, has emerged as a powerful architecture [16]. Taking advantage of an attention mechanism and positional encoding, Transformers is able to learn features from the relationship between two distant elements in sequential data, and thus have shown excellent performance in the analysis of serial data, e.g. natural language [16]. Its application has extended from natural language processing [17, 18] to fields such as image recognition [19], protein structure prediction [20], music generation [21], and image generation [22, 23], by treating data as sequential elements. Deep learning models have also made significant inroads into the medical domain [24, 25], including genetics [26, 27]. The CNN-based model DEEP*HLA was developed for HLA imputation [28]. DEEP*HLA represented a significant advancement in HLA imputation by facilitating more precise imputations than existing models. However, there was still room for improvement in DEEP*HLA, especially in accuracy for infrequent alleles, and its dependency on large size reference panels for effective imputation.

In this study, we introduce a Transformer-based model, named “HLA Reliable IMpuatioN by Transformer (HLARIMNT)”, designed to impute eight classical HLA alleles. HLARIMNT leverages the Transformer’s capability to exploit the sequential nature of SNPs. Our findings indicate that HLARIMNT consistently achieves higher imputation accuracy compared to CNN-based DEEP*HLA, showcasing the potential of Transformer-based models in enhancing the precision of HLA allele imputation.

## Materials and methods

### Datasets

We used Pan-Asian reference panel [29, 30] and Type 1 Diabetes Genetics Consortium (T1DGC) reference panel [31]. The Pan-Asian panel was genotyped with the Illumina HumanCoreExome BeadChip and T1DGC panel was genotyped using Illumina Immunochip, and both contain 4-digit resolution typing data of eight classical HLA genes based on SSO method; HLA-A, HLA-B, HLA-C, HLA-DRB1, HLA-DQA1, HLA-DQB1, HLA-DPA1, and HLA-DPB1. Both were publicly distributed with Beagle format in a phased condition. Pan-Asian reference panel is downloadable with SNP2HLA software (https://software.broadinstitute.org/mpg/snp2hla/). T1DGC panel is available from the NIDDK Central Repository after the registration process (https://repository.niddk.nih.gov/studies/t1dgc-special/). Pan-Asian reference panel contains 530 unrelated individuals, i.e., 1060 haplotypes, of Asian ancestry. T1DGC reference panel contains 5225 unrelated individuals, i.e., 10450 haplotypes, of European ancestry. The mixed panel was generated by combining all 530 individuals from Pan-Asian panel and randomly selected 530 individuals from T1DGC panel, resulting in a total of 1060 individuals. Number of all alleles and infrequent alleles (<1.0%) are shown in Table 1. Since all data used in this study have already been published and are publicly available, the ethics committee of our institute have waived an ethical review.

**Table 1:** Number of all and infrequent (<0.01) alleles

HLA	Pan Asian		T1DGC		Mixed panel	
	All alleles	Infrequent alleles	All alleles	Infrequent alleles	All alleles	Infrequent alleles
A	26	7	50	34	39	22
C	24	9	33	15	32	14
B	48	20	97	74	72	44
DRB1	32	11	51	33	48	28
DQA1	8	0	8	1	8	0
DQB1	15	1	18	5	18	3
DPA1	5	1	7	4	7	3
DPB1	21	6	34	20	27	16

### Model architectures

**Fig. 1:**
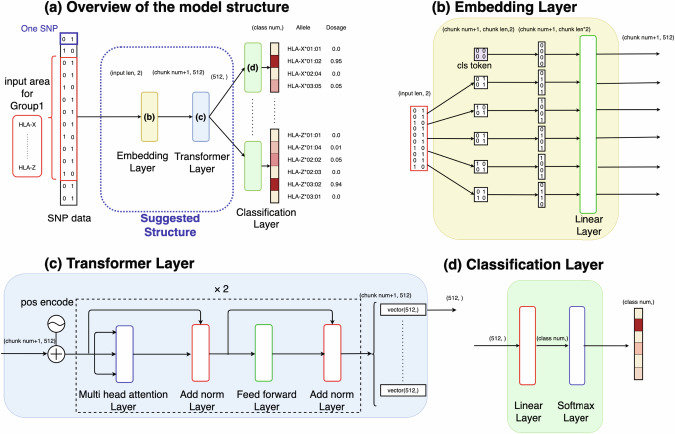
Architectures of HLARIMNT. **a** Overview of the model structure. **b** Embedding Layer. **c** Transformer Layer. **d** Classification Layer. HLARIMNT takes the input of each haplotype SNP genotype from pre-phased data represented as binary vectors, and outputs the genotype dosages of alleles for each HLA gene. input len: the number of SNPs to be input in Embedding Layer; chunk num: the number of chunks into with the SNPs are divided in Embedding Layer; chunk len: the number of SNPs in a single chunk; class num: the dimension of the output of Classification Layer

We adopted a basic structure of the model from DEEP*HLA and modified the CNN part of into Transformer-based architecture (the blue dotted line in Fig. 1, i.e., Embedding Layer and Transformer Layer).

HLA genes are divided into groups according to LD structure and physical distance as in DEEP*HLA; (1)HLA-A, (2)HLA-C, HLA-B, (3)HLA-DRB1, HLA-DQA1, HLA-DQB1 and (4)HLA-DPA1, HLA-DPB1, and genes in each group are imputed simultaneously. Therefore, the hyperparameters of the models are the same expect for the number of outputs, but the weights trained are different for each group. For each group, the model takes the input of each haplotype SNP genotype from pre-phased data, which are expressed by two-dimensional vectors. The SNPs are expressed by 01 or 10 based on whether each base is consistent with a reference or alternative one. The range of SNPs used for training in each group is the same as DEEP*HLA; 500kbps each.

Embedding Layer (Fig. 1b) consolidates the neighboring SNPs together to divide them into 50 chunks, and adds a classification (cls) token at the head of SNPs to learn the features. The cls token has the same shape as a single chunk, and its elements are all zeros. Then a common linear layer projects SNPs to the size of (51, 512).

Transformer Layer (Fig. 1c) applies positional encoding and the encoder portion of Transformer to the data, after which the feature vector of cls token is extracted.

Classification Layer (Fig. 1d), which is prepared for each HLA gene, is a combination of a linear layer and a softmax layer. Output dimension of the layer is the same as the number of alleles the HLA gene has. Output values are imputation dosages for the alleles, each of which takes a value from 0.0 to 1.0 that should be 1.0 when summed up.

To search hyperparameters, we utilized Oputuna (https://github.com/optuna). The searched parameters and the values are as follows; Number of Transformer heads: 64, Number of layer of Transformer encoder: 2, Dimension of feedforward in Transformer encoder: 64, Batch size: 64, Dimension of embedding in Embedding Layer: 512, Learning rate: 0.0005, and Number of epochs for early stopping: 50.

### Training

In the training, as with DEEP*HLA, we adopted hierarchical fine-tuning, in which the parameters for classifying 2-digit alleles were transferred to the model for 4-digit alleles. This allowed the model to take advantage of the hierarchical nature of HLA alleles. We only used SNP data for training and evaluation, and removed other information from the reference panel. We used Cross Entropy Loss as a loss function and Adam to optimize the loss. The Cross Entropy Loss is expressed by the following equation, whether it indicates correct output that should be 0 or 1, *y*_*i*_ indicates the output of softmax layer, and *n* indicates the number of classes.

$${Loss}= - \sum _{i=1}^{n}{t}_{i}\log {y}_{i}$$

The data was first split into test data and other data, then 90% of the other data was used as training data and 10% as validation data. The validation data was used for early stopping and model updates during the training.

First, a variant ‘count’, which is an indicator used for early stopping, is initialized to 0. At the beginning of the epoch, the training data is batched and trained using back propagation. At the end of one epoch, the model’s correctness rate (the percentage of data that the model could impute correctly) in the validation data is calculated. If this value is greater than the value of the ‘best model’, the weights are overwritten and saved as the ‘best model’. If not, add 1 to the ‘count’, and when the value of the ‘count’ reaches specified number, the training is terminated.

Also, at regular intervals, the learning rate is decreased. This process is repeated until the number of epochs exceed specified number, at which point training is terminated. The ‘best model’ stored at the end of training is used to examine the accuracy for the test data. For each gene, the average imputation accuracy was obtained by weighted average based on allele frequencies.

All experiments were conducted on a computer equipped CPU: AMD EPYC 7252 8-Core; memory: 125GB; and GPU: NVIDIA A5000 x1.

### Evaluation

In the experiments, we used 4 indices for the evaluations; *r*^2^, PPV, sensitivity, and probability, values of which were calculated for each allele.

*r*^2^(A) represents the square of Pearson’s product moment correlation coefficient between imputed and typed dosages and is expressed as follows;

$${r}^{2}(A)=\frac{{\bigg[{\sum }_{i=1}^{2n}{x}_{i}(A){y}_{i}(A)-\frac{\big({\sum }_{i=1}^{2n}{x}_{i}(A)\big)\big({\sum }_{i=1}^{2n}{y}_{i}(A)\big)}{2n}\bigg]}^{2}}{\Big({\sum }_{i=1}^{2n}{x}_{i}^{2}(A)-\frac{{\big({\sum }_{i=1}^{2n}{x}_{i}(A)\big)}^{2}}{2n}\Big)\Big(\Big({\sum }_{i=1}^{2n}{y}_{i}^{2}(A)-\frac{{\big({\sum }_{i=1}^{2n}{y}_{i}(A)\big)}^{2}}{2n}\Big)\Big)}$$

where *n* is the number of individuals (i.e., 2*n* is the number of haplotypes), *A* is the type of allele (e.g., HLA-A*01:23), *x*_i_(*A*) is the imputed dosage of allele A for haplotype *i*, which is obtained from the output of the softmax layer, and *y*_i_(*A*) is the typed dosage of allele *A* for haplotype *i*, taking values 1 if haplotype *i* has allele *A* and otherwise 0.

Sensitivity(*A*) represents the percentage of haplotypes that were correctly imputed to have allele *A* in all the haplotypes that have allele *A*, and PPV(*A*) represents the percentage of haplotypes with allele *A* in all the haplotypes predicted to have allele *A* (Naito 2021).

Probability(*A*) represents the imputed dosage of allele *A* for each haplotype with allele *A*, and is expressed as follows;

$${probability}(A)= \sum _{i=1}^{m}{x}_{i}(A)/m$$

where *m* represents the number of haplotypes with allele *A*.

Relevant codes are available at https://github.com/seitalab/HLARIMNT.

## Results

We first conducted a comparative analysis of imputation accuracy between CNN-based DEEP*HLA and Transformer-based HLARIMNT against three datasets: the Pan-Asian reference panel, the T1DGC reference panel, and the mixed panel derived from both.

After the training of the models, imputation was executed against the test dataset for 4-digit alleles for eight HLA genes. Accuracy for each allele was assessed using four indices; *r*^2^, PPV, sensitivity, and probability. This training-test session was repeated 5 times individually, then calculated the simple additive average of each index to compare the two methods.

**Fig. 2:**
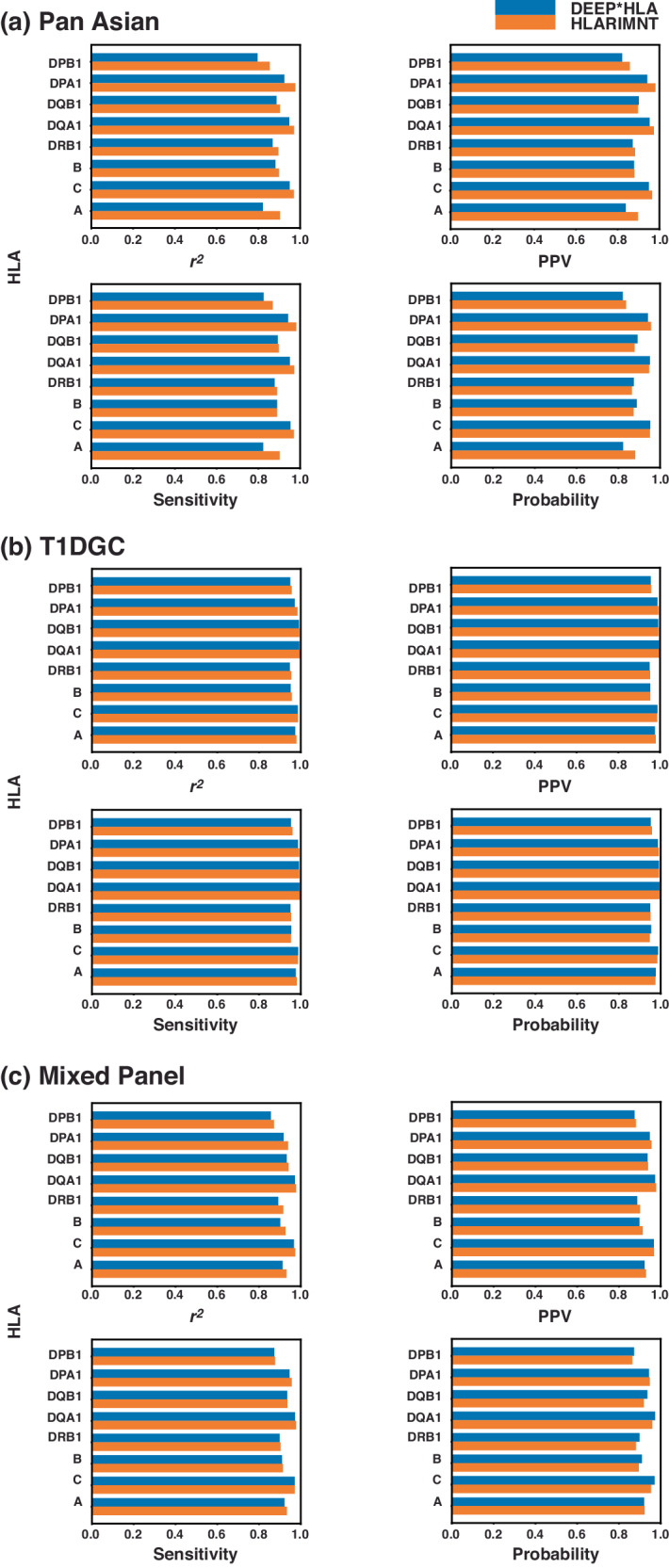
Average accuracy for 4-digit alleles in each of the HLA genes. **a** Pan Asian reference panel, **b** T1DGC reference panel, and **c** Mixed reference panel. HLARIMNT outperformed DEEP*HLA on most genes and indices for all data datasets, though there were some exceptions depending on the combination of indices and genes

Imputation accuracy for each HLA gene is shown in Fig. 2. Generally, HLARIMNT demonstrated superior performance for nearly all genes across the three reference panels, with certain exceptions based on the specific gene-reference panel-index combinations.

Table 2 is the summary of 5 experiments. HLARIMNT demonstrated superior accuracy across all indices for all 4-digit alleles across the reference panels, albeit DEEP*HLA exhibited equal Probability in the T1DGC reference panel and slightly higher Probability in the Mixed reference panel. Imputation accuracy for infrequent 4-digit alleles (with frequencies less than 0.01) was also tested using T1DGC panel (Table 3). The size of test data of the other panels were too small to test infrequent alleles. Table 3 highlights HLARIMNT’s enhanced performance for these alleles compared to its performance across all alleles.

**Table 2:** Average imputation accuracy for all alleles (*n* = 5)

Dataset	Indices	DEEP*HLA		HLARIMNT	
		Mean	S.D.	Mean	S.D.
Pan-Asian	r^2^	0.884	0.024	**0.922**	0.015
	PPV	0.894	0.023	**0.917**	0.020
	Sensitivity	0.895	0.023	**0.922**	0.015
	Probability	0.893	0.023	**0.899**	0.021
T1DGC	r^2^	0.970	0.004	**0.976**	0.003
	PPV	0.974	0.003	**0.976**	0.003
	Sensitivity	0.974	0.003	**0.977**	0.003
	Probability	**0.974**	0.004	**0.974**	0.003
Mixed	r^2^	0.919	0.004	**0.935**	0.003
	PPV	0.926	0.003	**0.933**	0.003
	Sensitivity	0.929	0.003	**0.934**	0.003
	Probability	**0.928**	0.004	0.918	0.003

The best result of each index is highlighted in bold

**Table 3:** Average imputation accuracy for infrequent (<0.01) alleles (*n* = 5)

Dataset	Indices	DEEP*HLA		HLARIMNT	
		Mean	S.D.	Mean	S.D.
T1DGC	r^2^	0.860	0.030	**0.880**	0.022
	PPV	0.893	0.047	**0.900**	0.028
	Sensitivity	0.846	0.043	**0.860**	0.036
	Probability	0.846	0.040	**0.853**	0.037

The best result of each index is highlighted in bold

**Fig. 3:**
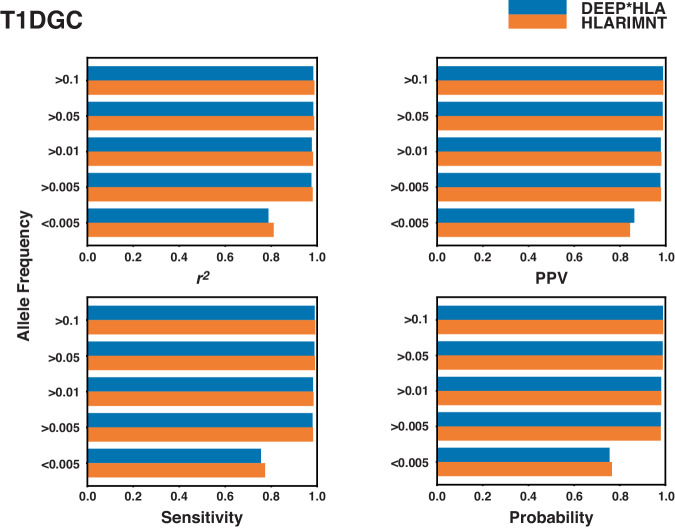
Average values of the 4 indices calculated for each allele frequency. At almost all of the frequencies, the accuracy of HLARIMNT was equal to or better than DEEP*HLA. Furthermore, the advantage of HLARIMNT was more noticeable at lower frequencies

The influence of allele frequency on imputation accuracy was further examined using the T1DGC panel (Fig. 3). This analysis displayed the accuracy for 4-digit alleles with frequencies at or above the threshold indicated on the vertical axis, plotted against the horizontal axis (noting that <0.005 represents a frequency of less than 0.005). Regardless of the allele frequencies, HLARIMNT achieved comparable or superior accuracy to DEEP*HLA.

**Fig. 4:**
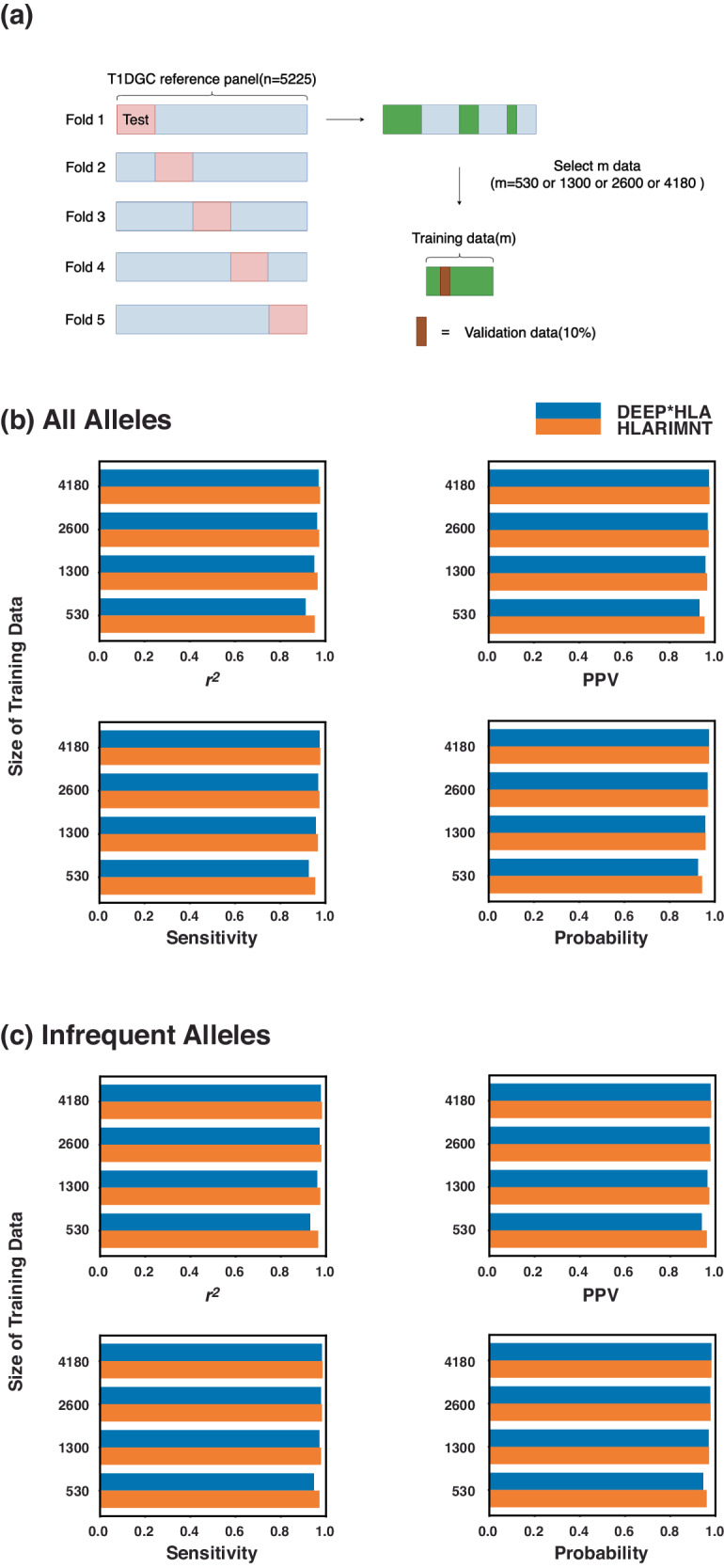
**a** Preparation of small size training data. **b** The average accuracy for 4-digit alleles using various sizes of training data and (**c**) shows that of alleles which have frequencies less than 0.01. The accuracy of HLARIMNT was generally higher than that of DEEP*HLA on any training data sizes

Subsequently, we explored the effect of training data size using the T1DGC reference panel (Fig. 4). The training dataset comprised various subsets of individuals, including sizes of 530, 1300, 2600, and 4180, randomly selected from the T1DGC reference panel. The test dataset remained constant and independent across all training data sizes. Figure 4b shows the average accuracy for all 4-digit alleles across different training data sizes. HLARIMNT consistently achieved equal or higher accuracy than DEEP*HLA across all sizes of training data. Moreover, the advantage of HLARIMNT over DEEP*HLA was more pronounced with smaller training data sizes across all indices. For infrequent alleles (with frequencies less than 0.01), HLARIMNT exhibited superior performance across all sizes of training data, as shown in Fig. 4c. Similar to previous findings, HLARIMNT’s advantage was more significant with smaller training datasets. This trend suggests that HLARIMNT’s effectiveness is particularly notable when the available training data is limited, underscoring its efficiency in imputing HLA alleles, especially those that are infrequent.

## Discussion

A significant observation from our study is that HLARIMNT demonstrated superior performance in a mixed panel combining two ethnic groups, suggesting its capability to capture features of the SNPs without overfitting to a specific ethnic background. This attribute is particularly beneficial for HLA imputation, which typically requires large reference panels for accuracy. In fact, both methods in this paper were more accurate with a larger number of training data. On the other hand, it is known that the distribution and frequency of the HLA alleles are variable across different ethnic groups [32], which results in heterogeneity in HLA risk alleles across populations [30]. This phenomenon is seen, for example, in the association between non-Asp57 in HLA-DQB1 and type 1 diabetes (T1D) risk; in Europeans, there is a strong correlation between these two [33, 34], but not in Japanese [35]. Therefore, it is desirable to create large reference panels for each race. However, to create large reference panel, it is necessary to analyze SNPs of many individuals at high density, which is very expensive. In this regard, if we can use the mixture of reference panels including various ethnic groups for training, it will be possible to perform an accurate HLA imputation without performing new SNP sequencing to make the reference panel larger. HLARIMNT has the potential to meet this requirement.

Our results further underscore HLARIMNT’s enhanced accuracy with smaller datasets, irrespective of allele frequencies. Although haplotypes vary by ethnicity—necessitating ethnically specific reference panels—the financial implications of creating new panels for each ethnicity are considerable. HLARIMNT’s efficacy in accurately capturing characteristics with less data offers a potential solution to this problem, positioning it as a viable deep learning method for HLA imputation. This study aligns with recent research highlighting the Transformer model’s utility in processing sequential data, suggesting its superiority over traditional methods like RNNs and CNNs in genomic applications. This finding invites further exploration of Transformer models within genomics.

However, several aspects warrant discussion, including the limited size of the Pan-Asian reference panel and the potential unreliability of accuracy for alleles with very low frequencies. A detailed analysis of these errors could illuminate the model’s allele differentiation process and guide improvements in imputation accuracy. Further validation of HLARIMNT’s utility could involve testing with data from different ethnicities or examining accuracy variations among SNP sequences. Such an approach would necessitate careful selection of closely related ethnic groups for training and testing, given the significant allele frequency differences across ethnicities.

The study demonstrates HLARIMNT’s superior accuracy across all reference panels, notably for infrequent alleles, and its consistent performance across various training data sizes. These findings suggest HLARIMNT’s capacity for efficient HLA imputation across diverse populations and data volumes, highlighting its potential as a practical tool in the field.
